# Therapeutic Potential of Gut Microbiota and Its Metabolite Short-Chain Fatty Acids in Neonatal Necrotizing Enterocolitis

**DOI:** 10.3390/life13020561

**Published:** 2023-02-16

**Authors:** Naser A. Alsharairi

**Affiliations:** Heart, Mind and Body Research Group, Griffith University, Gold Coast, QLD 4222, Australia; naser.alsharairi@gmail.com

**Keywords:** necrotizing enterocolitis, premature infant, short-chain fatty acids, metabolites, gut microbiota

## Abstract

Short chain fatty acids (SCFAs), the principle end-products produced by the anaerobic gut microbial fermentation of complex carbohydrates (CHO) in the colon perform beneficial roles in metabolic health. Butyrate, acetate and propionate are the main SCFA metabolites, which maintain gut homeostasis and host immune responses, enhance gut barrier integrity and reduce gut inflammation via a range of epigenetic modifications in DNA/histone methylation underlying these effects. The infant gut microbiota composition is characterized by higher abundances of SCFA-producing bacteria. A large number of in vitro/vivo studies have demonstrated the therapeutic implications of SCFA-producing bacteria in infant inflammatory diseases, such as obesity and asthma, but the application of gut microbiota and its metabolite SCFAs to necrotizing enterocolitis (NEC), an acute inflammatory necrosis of the distal small intestine/colon affecting premature newborns, is scarce. Indeed, the beneficial health effects attributed to SCFAs and SCFA-producing bacteria in neonatal NEC are still to be understood. Thus, this literature review aims to summarize the available evidence on the therapeutic potential of gut microbiota and its metabolite SCFAs in neonatal NEC using the PubMed/MEDLINE database.

## 1. Introduction

The gut microbiota composition and function undergo drastic changes during the first years of life [1] that are characterized by early microbial colonization with *Escherichia* and *Bifidobacterium*, which are gradually decreased following weaning and replaced by species of obligate anaerobic bacteria within the Firmicutes phylum, such as *Coprococcus, Enterococcus, Roseburia* and *Clostridium* [2,3,4]. The short-chain fatty acids (SCFAs), butyrate, propionate and acetate, are the main metabolic products of gut microbial Firmicutes phyla fermentation of complex non-digestible dietary substrates, such as fiber and resistant starch [5]. *Bifidobacterium* and lactic acid bacteria (LAB) produce lactate, which acts as an intermediate fermentation product for butyrate production [6]. In general, *Bifidobacterium* spp. are considered the primary colonizers of the breastfed infant gut, mainly due to the presence of human milk oligosaccharides (HMOs) [7,8,9,10]. The degradation of HMOs and complex carbohydrates (CHO) could result in high SCFA production (mainly butyrate and acetate), which can be used by other butyrogenic bacteria, such as *Faecalibacterium prausnitzi* (*F. prausnitzi*), *Bacteroides* and *Roseburia,* for growth by cross-feeding [7,8]. SCFAs produced by the infant-type *Bifidobacterium* spp. can enhance immunomodulatory responses by reducing inflammatory cytokines through the microbiota-gut-brain axis [10].

Necrotizing enterocolitis (NEC) is a severe inflammatory necrosis of the distal small intestine/colon that primarily affects preterm (less than 32 weeks’ gestation) or very low birth weight (VLBW: <1500 g) infants after the introduction of enteral feeds. NEC is characterized by hyperosmolar injury and intestinal ischemia, which reduce the integrity of the epithelial barrier that is evident by peritonitis, hemodynamic instability, abdominal tenderness/cellulitis, acute feeding intolerance, bacteremia and abdominal distension [11,12]. The aetiology of NEC is not clear, but it is thought to be related to several factors, including pre-eclampsia, aberrant bacterial colonization (e.g., infection), premature rupture of the membranes, placental abruption, intrauterine growth restriction, LBW, patent ductus arteriosus, sepsis and anemia [13]. Early gut microbial dysbiosis is also implicated in disease pathogenesis, in which the gut microbiota composition of preterm infants with NEC is characterized by reduced abundances of *Bifidobacterium*, *Firmicutes* and *Bacteroidetes* and increased abundances of *Prevotella*, *Clostridioides*, *Staphylococcaceae*, *Proteobacteria*, *Enterobacteriaceae*, *Rothia*, *Streptococcus* and *Blautia* [14,15,16,17,18,19,20].

Pregnancy and lactation perform a crucial role in shaping the composition of infant gut microbiota, which is influenced by a range of pre-and post-natal factors, such as antibiotic exposure, lactation stage, gestational age, mode of feeding/delivery, diet and body mass index (BMI) [3,17,21]. Maternal diet during pregnancy and lactation has been linked to an increased risk of developing obesity and asthma in the infant’s early years of life [22,23] and the mechanisms underlying such effects are postulated to be the alterations in maternal/infant gut microbiota and/or milk microbiota [21]. A high-fiber diet during pregnancy and lactation increases SCFAs production [21,22,23,24,25,26]. SCFAs are essential for differentiation of helper T cells (Th1, Th2) by their binding to G-protein coupled receptors (GPCRs), including free the fatty acid 2/3 receptor (FFAR2, FFAR3) present in the colon, thereby maintaining gut homeostasis and regulating inflammation by reducing the expression of pro-inflammatory cytokines [24,25]. Higher levels of SCFAs detected in breastmilk may enhance the neonatal anti-inflammatory immune responses by inducing factor fork head box protein 3 (FOXP3^+^) regulatory T (T_reg_) cell differentiation in the gut [27]. Breastmilk is a source of secretory IgA immunoglobulin A (SIgA) and IgA-producing antibody-secreting cells (ASCs), which regulate early gut microbiota maturation and immunity by binding to SCFA-producing *Bifidobacterium* and *Lactobacillus*, resulting in reduced NEC-related inflammation in preterm infants [28]. It has been suggested that infant feeding with probiotics-supplemented formulas and solid/complementary foods alter gut microbiota composition during the first years of life [9,29,30,31]. Evidence from randomised controlled trials (RCTs) has shown that NEC-specific treatments, such as oral lactoferrin combined with probiotics and parenteral/oral supplementation with arginine, reduce the disease risk [13,32,33,34,35]. Probiotic supplementation with *Bifidobacterium* and *Lactobacillus* strains, prebiotics (e.g., HMOs), synbiotics (mixtures of probiotics and prebiotics), long chain polyunsaturated fatty acid (PUFA) and bovine colostrum were also demonstrated by a large number of human RCTs, to be effective preventive strategies for NEC, which are thought to modulate the immune response and increase the abundance of beneficial gut microbes [36,37,38,39]. Breastfeeding has been demonstrated to have a protective role against NEC due to its potential to promote the colonization of commensal bacteria and decrease the susceptibility to gut dysbiosis in premature infants [40,41].

SCFAs contribute as mechanisms linking diet, gut microbiota and human health [5], resulting in induced epigenetic changes in the gene patterns of offspring, thereby being a potential epigenetic target in the treatment of gastrointestinal diseases during the first years of life. SCFAs could alter DNA and histone methylation patterns in several genes, resulting in reduced cytokines and chemokines with pro-inflammatory effects in the infant’s gut [22,23]. Findings from recent reviews in infants/children have demonstrated the potential efficacy of SCFA-producing bacteria in reducing inflammation-related disease risk, including obesity, asthma and inflammatory bowel diseases (IBD) [22,23,42]. However, no reviews yet discuss the role of SCFAs and SCFA-producing bacteria as therapeutic agents against neonatal NEC. Thus, this review aims to explore the therapeutic role of gut microbiota and its metabolite SCFAs in neonatal NEC. It is hypothesized that gut microbial-derived SCFA metabolites can be regarded as having health benefits in neonatal NEC. Preterm infants with NEC who are fed breastmilk/formula and/or supplemented with probiotics/prebiotics are postulated to have higher SCFA levels and abundance of SCFA-producing bacteria, which may perform a significant role in modulating the inflammatory immune responses of immature intestinal cells.

## 2. Methods

A literature search of PubMed/MEDLINE database was performed up to December 2022 to identify studies exploring the potential role of gut microbiota and its metabolite SCFAs in NEC treatment using the following keywords “NEC”, “preterm/premature infants”, “immature intestinal cells”, “intestinal inflammation”, “inflammatory biomarkers”, “gut microbiota”, “epigenetic”, “SCFAs”, “probiotics/prebiotics” and “feeding types”. The search was limited to retrieve human studies published in English irrespective of design.

## 3. Epigenetics and Inflammatory Biomarkers in NEC

Epigenetic alternation in the immature intestine, such as changes in DNA methylation and long non-coding RNA (lncRNA) patterns, may contribute to increased risk of NEC. Epigenetic changes are attributed to prenatal and postnatal factors (e.g., microbiome, intrauterine infection and enteral feeding) that may affect intestinal function/structure and cause upregulation of pro-inflammatory cytokines [43,44]. DNA methylation changes in cytosine-phosphate-guanine dinucleotides (CpG) regions of NEC-related genes are related to disease risk. For example, high levels of CpG methylation in the *DNMT3A*, *TNT2/3*, *TNIP1*, *GALNT6* and *HNF4* genes have been identified in stools and colons of premature infants with NEC [45,46,47]. An association of CpG methylation in the cytokine Oncostatin M (OSM) with NEC has also been observed, which can induce intestinal inflammation [47]. The hypermethylation of four genes (*MPL*, *KDM6A*, *ZNF335* and *RASAL3*) has been reported in the intestine of neonatal NEC, which is associated with lymphocyte proliferation and intestinal epithelial permeability [48]. Analyses of CpG methylation positions in the intestinal epithelial cells of neonatal NEC revealed a significant hypermethylation in five genes (toll-like receptor 4; TLR4, *ENOS*, *EPO*, *DEFA5* and *VEGFA*) at three sites [49]. Overexpression of micro-431 (miR-431) in the intestinal tissues of neonatal NEC results in significantly inhibited *FOXA1* and *HNF4A* and increased pro-inflammatory (e.g., interleukins IL-6, IL-8, IL-10, *LGR5*, tumor necrosis factor-α; TNF-α and *PRKCZ*) gene expression in response to lipopolysaccharide (LPS) stimulation [50]. lncRNA influences the expression of mRNAs in the intestine tissues of neonatal NEC by upregulating expression levels of IL-6, IL-1β and TLR4 after LPS exposure, which induces activation of peroxisome proliferator-activated receptors (PPARs) and phosphatidylinositol-3 kinase/serine-threonine kinase (PI3K-AkT) signaling pathways, suggesting that lncRNA contributes to NEC pathogenesis [51].

NEC is characterized by decreased FOXP3^+^ T_reg_ cell levels and gut expression of transforming growth factor β (TGF-β). Infants with NEC displayed elevated levels of nitric oxide (NO) and high cytokine expression levels with pro-inflammatory effects (e.g., Nuclear factor-_κ_B; NF-_κ_B, tumor necrosis factor-α; TNF-α, interferon; IFN-γ, IL-6, IL-8, IL-10, IL-1β) induced by LPS and produced by the cells of the adaptive immune system in response to colonization by pathogenic bacteria (e.g., *Staphylococcus* spp., and *Clostridium* spp.), thereby disturbing the integrity of epithelial tight junctions [52,53,54,55,56,57,58]. An experimental study has shown overexpression of TLR2 and TLR4 receptor-mediated IL-8 mRNA expression in the immature intestine of neonatal NEC [59]. Data from a human NEC experiment showed that pro-inflammatory cytokine expression of IL-1β, IL-1A, IL-6, TNF-α and IL-36 isoforms IL36A were increased in epithelial cells, whereas cytokines IL-37 and IL-22, which are considered protective, were decreased [60]. Evidence from an experimental study showed that IL-17F expression and its related pro-inflammatory C-X-C motif chemokines ligand 8 and 10 (CXCL8, CXCL10) are upregulated in the intestine of neonatal NEC [61]. A case–control study demonstrated higher levels of TNF-α, IL-8, IL-1β and lower levels of TGF-β, FOXP3^+^ T_reg_ and IL-10 in the ileum of surgical NEC patients compared with matched controls (patients with spontaneous intestinal perforation/congenital intestinal atresia) [18]. Another case–control study showed that the levels of serum TNF-α, IL-6 and intestinal fatty acid-binding protein (I-FABP) were higher in NEC patients than non-NEC counterparts [62]. In a recent experimental study, preterm newborns displayed increased mRNA expression of fecal cytokines IL-1α/β, IL-7 and IL-12p40 [20]. This suggests that preterm infants with NEC display intestinal inflammation with markedly increased pro-inflammatory cytokines and chemokines, in which DNA methylation and lncRNA as epigenetic mechanisms are involved.

## 4. Insights into the SCFA-Producing Bacteria in Preterm Infants

SCFAs produced by gut microbiota act as epigenetic mechanisms in reducing intestinal inflammation by inducing DNA/histone methylation changes in the gene patterns of infants [22,23,42]. On this basis, it is important to provide an overview of SCFA-producing bacteria, such as *Bifidobacterium*, *Lactobacillus*, *Enterococcus* and *Bacteroides,* that may perform a crucial role in protecting from NEC-related inflammation.

The role of SCFAs in neonatal NEC treatment remains controversial. High luminal SCFAs production (e.g., butyric acid) by bacterial colonization in preterm infants is due to poor gastrointestinal motility and carbohydrates malabsorption [63]. Enteric bacterial pathogens, including *Clostridium perfringens* (*C. perfringens*), *C. difficile*, *C. paraputrificum*, *C. butyricum* and *Klebsiella pneumoniae* (*K. pneumoniae*) have shown to be implicated in NEC via increasing butyric acid production as result of lactose fermentation [64]. A high production of butyric acid by *C. butyricum* increases inducible nitric oxide synthase (iNOS) gene expression responsible for mucosal injury in NEC [65]. These findings suggest that excessive SCFAs produced by pathogenic bacteria may reduce the intestinal epithelial barrier integrity and increase metabolic inflammation in neonatal NEC. Thus, it is proposed that SCFA-producing commensal microbes (e.g., *Bifidobacterium*, *Lactobacillus*) may contribute to the regulation of gut immune homeostasis.

Preterm infants demonstrated a significantly less diverse microbiome, including *Bifidobacterium* spp. [14,15,16,17,18,19,20]. *Bifidobacterium* spp. within the Actinobacteria phylum are Gram-positive non-motile/spore forming bacteria [66,67] dominated in the gut of breastfed infants [10]. Several strains belonging to *Bifidobacterium* spp., including *B. breve, B. longum* subsp. *longum*, *B. longum* subsp. *infantis* and *B. bifidum*, are among the prevalent members of breastmilk [68,69,70,71,72,73]. The genomes of *Bifidobacterium* strains include a large set of enzymes belonging to the glycosyl hydrolase family (β-N-acetylhexosaminidase, *α-L-fucosidases*) essential for HMO degradation in the breastfed infant gut via the intracellular galacto-N-biose/lacto-N-biose (GNB/LNB) pathway [70,74,75]. HMOs, such as galacto-oligosaccharide (GOS) and fructo-oligosaccharide (FOS), may act as prebiotics, which perform a key role in the development of the infant gut microbiota [76]. FOS promotes the growth of *B. breve* and *B. bifidum* in preterm fecal microbiota, which produce high levels of butyric, propionic and acetic acids [77]. Fermentation of resistant starch by *Bifidobacterium* spp. has been shown to increase production of acetate, propionate and butyrate in pre-weaning and weaning infant feces [78].

*Lactobacillus* is a genus of Gram-positive facultative LAB that is typically classified in the class Bacilli, phylum Firmicutes [66,79]. *Lactobacillus* spp. were detected at a relatively high percentage in the meconium of preterm infants [80,81]. Breastmilk and the breast-fed infant gut are dominated by several *Lactobacillus* spp. mainly *L. rhamnosus*, *L. gasseri*, *L. reuteri*, *L. acidophilus*, *L. fermentum*, *L. crispatus*, *L. paracasei* and *L. salivarius* [68,69,82,83,84,85]. *Lactobacillus* spp. produce high D(−), L(+) and DL-Lactic acid levels [86], which have the ability to generate SCFAs, bacteriocins and FOS [87,88].

Analysis of the fecal and meconium microbiota of preterm infants revealed a high abundance of *Enterococcus* [80,89,90]. *E. faecalis* and *E. faecium* constitute the most dominant *Enterococcus* of fecal microbiota in preterm infants [80]. The genus *Enterococcus* belongs to a large group of LAB in the class Bacilli that is typically identified as facultative anaerobic bacteria within the Firmicutes phylum [66,79,91]. *Enterococcus* spp. produce L(+)-Lactic acid as the main end metabolic product yielded from sugar fermentation [86]. *Enterococcus* increases the production of acetate, propionate and butyrate in pre-weaned and weaning infants’ feces upon fermentation of resistant starch [78]. The *E. faecalis* strain ATCC19433 exhibits growth in response to fucosylated HMOs (2′-FL or 3-FL) and produces lactate [92]. The *E. faecalis* strain AG5 has been found to assimilate cholesterol and produce propionate in vitro [93].

Multiple *Streptococcus* spp. (*S. thermophilus*, *S. mitis*, *S. anginosus* and *S. sanguinis*) dominated the meconium microbiome of preterm infants during the first 21 days of early life [80]. The genus *Streptococcus* is a facultative anaerobe Gram-positive bacteria within the Firmicutes phylum [94]. *S. thermophilus* has been shown to produce L(+)-Lactic acid as the major fermentative end-product [86]. Such species can use the acetyl-CoA “Wood-Ljungdahl” pathway of carbon dioxide (CO2) fixation as the main mechanism for transforming pyruvate to acetate [95]. Fucosylated HMOs (2-FL or 3-FL) are found to be metabolized into lactate by the *S. thermophilus* strain ATCC19258 [92].

The genus *Bacteroides* that belongs to the phylum Bacteroidetes, is the most abundant Gram-negative anaerobic bacteria in the fecal microbiota of preterm infants [96]. *B. fragilis* was the species that predominated in the fecal microbiota of preterm infants in the first weeks of life [97,98]. *Bacteroides* spp. have large genomes with extremely high numbers of carbohydrate cleaving enzymes [99,100,101], which degraded complex oligosaccharides, such as mucin glycans [102] and HMOs [103,104,105]. Butyrate, acetate and propionate were the major end-products of resistant starch fermentation generated by *Bacteroides* spp. in weaned infants’ feces [78]. The acetyl-CoA and succinate pathways are the major routes for the production of acetate and propionate, which exist mainly in *Bacteroides* spp. [95,106,107,108].

## 5. Effects of Feeding Types on Gut Microbiota and Its Metabolite SCFAs in Preterm Infants

Evidence from several prospective cohort studies and RCTs suggests that breastmilk and/or formula with probiotics/prebiotics may have the potential to enhance the growth of SCFA-producing bacteria and increase SCFAs levels in preterm infants.

### 5.1. Prospective Cohort Studies

A cohort study over 1-year period has demonstrated the potential of breastmilk and formula to influence fecal SCFA profiles in LBW preterm infants. The concentrations of fecal acetate and propionate were found to be higher in infants who were fed breastmilk, whereas the concentrations of fecal butyrate were higher in those fed Similac special care formula [109]. It has been has shown that premature infants fed formula have higher concentrations of butyrate and acetate, while those fed breastmilk have higher concentrations of propionate in their feces over 1-year follow-up. This may be due to the colonization of SCFA-producing *Bifidobacterium* and *Lactobacillus* influenced by breastmilk and formula [110]. A study evaluated the beneficial effects of probiotics supplementation in preterm infants over 100-day period and found that a combination of *B. bifidum* with *L. acidophilus* (Bif/Lacto) increases fecal lactate levels and the abundance of *Bifidobacterium* spp. consistent with their ability to metablize HMOs into acetate [111].

Over a 10-year longitudinal study, preterm infants who were fed breastmilk or supplemented with two different probiotics (Infloran and Labinic) showed a significant increase in the relative abundance of *Bifidobacterium* spp. and a significant decrease in the relative abundance of pathogenic bacteria in their feces. The study suggests that long-term colonization of *Bifidobacterium* depends on the type of probiotics used [112]. In a recent study, with follow-up over 1 year, aimed to identify variation in fecal microbiota from admission to discharge, preterm infants who were fed breastmilk demonstrated a higher abundance of *Bifidobacterium* spp. and lower abundance of *Veillonella*. However, infants who were fed probiotic formula demonstrated a lower abundance of *Lactobacillus* [113]. A cohort study showed that the gut microbiota colonization varied among preterm infants as a result of probiotic formula-feeding. *Bifidobacterium* and *Lactobacillus* were found in higher abundance in the fecal microbiota of preterm infants fed with formula supplemented with probiotic *B. lactis* over 3-month period [114]. Use of the probiotic *B. longum* subsp. *infantis* EVC001 in conjunction with breastmilk has been shown to increase the gut microbiome abundance of Bifidobacteriaceae and decrease the abundance of Staphylococcaceae and Enterobacteriaceae associated with gut dysbiosis and antibiotic-resistance in preterm infants in longitudinal 5 months of follow-up [115]. The relative abundances of *Bifidobacterium* spp. have been found to increase the fecal microbiota of preterm infants fed with breastmilk and human donor milk during the first three months of life [116]. In a study conducted to examine the effect of breastmilk, donor human milk or formula on shaping the fecal microbiota of preterm infants during the first month of life, infants fed with mother’s own milk have higher fecal SCFA-producing bacteria compared with those fed donor human milk or formula [117]. A previous study found that breastmilk influences the microbial colonization of preterm infants over the first 30 days of life. Breastmilk fed infants have higher abundance in *Lactobacillus* and *Granulicatella* than non-breast milk fed infants [118].

A study found that oral administration of *B. breve* M-16V to LBW infants resulted in increased fecal abundance of *Bifidobacterium* and *Enterococcus* 10 weeks post-administration. This is attributed to acetic acid, which may inhibit the growth of Proteobacteria, thus providing a suitable environment for SCFA-producing bacteria growth [119]. Evidence from a cohort study supports supplementation with multiple-strain probiotics including bifidobacteria in preterm infants. Three *Bifidobacterium* strains have been administrated in infants showing lower detection rates of Enterobacteriaceae and higher rates of bifidobacteria in the feces over 6-month follow-up [120]. A longitudinal multi-center study has shown that the fecal microbiota of preterm infants after supplementation with probiotic Infloran have a high relative abundance of *Bifidobacterium* and *Lactobacillus* up to 4 months of age [121]. Another multi-center cohort study showed that administering a combination probiotics mixture to preterm infants resulted in influenced the fecal microbiota profile with *Lactobacillus* and *Bifidobacterium* predominating over 5-month period [122]. In a cohort study over a 5-month follow-up, suspected bacterial signatures from *Bifidobacterium* and *Lactobacillus* were identified in the fecal microbiota of preterm infants after discontinuation of a probiotic mixture containing *Bifidobacterium* and *Lactobacillus* spp. [123].

In one cohort study, the administrated strain, *B. bifidum* ATCC15696 and *L. acidophilus* NCIMB701748, showed significant alterations in the fecal microbiota of preterm infants as demonstrated by high *Lactobacillus* and *Bifidobacterium* abundances over 1-month period [124]. A cohort study on human milk-fed preterm infants has indicated probiotic supplementation with *B. longum* subsp. *infantis* influences the fecal colonization by *Bifidobacterium* [125]. Probiotic supplementation with *L. rhamnosus* GG and *B. animalis* ssp. *lactis* BB-12 increases the relative abundance of Firmicutes and Actinobacteria and decreases the abundance of *Weissella*, *Veillonella* and *Klebsiella* in preterm infants during the first month of life [126]. A recent study showed that supplementation of probiotics rich in *L. acidophilus* and *B. bifidum* alters the fecal microbiota of preterm infants during the first 30 days of life by increasing *Lactobacillus* spp. and *E. faecium* abundances [127].

### 5.2. RCTs

A previous study resulted in increased fecal colonization with bifidobacteria and acetic acid levels in premature infants after receiving formula supplemented with prebiotic/probiotic combinations compared to those in the placebo group [128]. In a recent study, multi-strain probiotics are found to be effective in increasing fecal butyric and propionic acid levels, whereas single-strain probiotic increases fecal butyric acid levels only in preterm infants. Infants who were supplemented with probiotics showed higher fecal abundance of *Bifidobacterium* spp. and lower fecal abundance of *Clostridium* [129]. An RCT showed that preterm infants receiving probiotic supplementation with the *B. breve* (BBG-01) strain in conjunction with breastmilk and feeding with maternal colostrum have higher fecal *Bifidobacterium* abundance compared with those in placebo groups [130]. In one previous study, supplementation of a bovine milk formula with galacto and fructo-oligosaccharide mixtures has been shown to increase the relative abundance of fecal bifidobacteria in preterm infants [131]. Preterm infants supplemented with a probiotic mixture containing *S. thermophilus* TH-4, *B. longum* subsp. *infantis* BB-02 and *B. animalis* subsp. *lactis* BB-12 showed a significant increase in the relative abundance of *Bifidobacterium* spp. in the gut microbiota compared to those in the placebo group [132]. Supplementation of preterm infants with the *B. lactis* Bb12 probiotic strain compared with placebo modulates the gut microbiota by lowering the cell counts of clostridia and enterobacteria and increasing the cell count of bifidobacteria [133]. Probiotic supplementation with *L. reuteri* DSM 17938 modulates the gut microbiota composition in preterm infants by increasing the relative abundance of *Lactobacillus* and decreasing the abundance of Clostridium, Enterobacteriaceae and Staphylococcaceae [134]. A supplementation with a mixture of *S. thermophilus* TH-4, *B. animalis* subsp. lactis BB-12 and *B. longum* subsp. *infantis* BB-02 increases the bacterial abundance of probiotic species in the preterm infant’s gut [135].

The effects of feeding types on fecal SCFAs and SCFA-producing bacteria in preterm infants are summarized in Table 1.

## 6. Role of Gut Microbiota and Its Metabolite SCFAs as Therapeutic Potential Agents in NEC

Gene expression including cytokines and chemokines result from histone and long non-coding RNA (lncRNA) modifications have been linked to immune cell function and inflammation in NEC [45,46,47,48,49,50,51]. Given that SCFAs have been identified as epigenetic modifier exert anti-inflammatory effects in inflammatory diseases [22,23,42], it is likely that SCFA-producing bacteria and SCFAs could perform an epigenetic role in modulating immune responses in the inflamed gut of neonatal NEC by reducing pro-inflammatory cytokines and chemokines. Thus, this section presents the therapeutic role of gut microbiota and its metabolite SCFAs in regulating NEC-related inflammation in which epigenetic changes are implicated.

Evidence from a few studies has revealed a significant decrease in the levels of butyric, propionic and acetic acids in NEC patients [18,19]. It has been shown that colonization of fecal microbiota of NEC patients with *Firmicutes* and *Bacteroidetes* increases butyric acid synthesis, resulting in increased T_reg_/T_helper_ cell ratio [18]. An in vitro study showed that butyrate inhibits IL-1β-induced IL-6, CX3XL1 and CXCL5 gene expression in human immature enterocytes (H4 cells) and regulates tight junction and mucin-related gene expression via increasing the mRNA expression of Mucin (MUC20), Claudins (CLDN4, CLDN11 and CLDN15) and Occludin (OCLN) [136]. In fetal small intestinal epithelial FHs 74 Int cells, butyrate, acetate and propionate were found to decrease IL-1β-induced IL-6 and IL-8 mRNA levels through inhibiting the activation of extracellular signal-regulated kinase 1/2 (ERK1/2), c-JUN NH2-terminal kinase 1/2 (JNK1/2) and NF-_κ_B p65 signaling pathways [137]. Treatment of fetal immature enterocytes (H4 cells) with butyrate, acetate and propionate results in a significant inhibition of IL-1β-induced histone deacetylase 3 and 5 (HDAC3, HDAC5) and IL-8 mRNA expression and activation of G-protein coupled receptor 109A (GPR109A) mRNA expression [24]. Butyrate and propionate have been shown to reduce inflammation in vitro by inhibiting several chemokines (e.g., CCL3, CCL4, CCL5, CCL9) and LPS-induced IL-6 and IL-12p40 mRNA expression in both mature and immature human monocyte-derived dendritic cells (DCs) [138]. In vitro treatment of amnion epithelial and mesenchymal cells in preterm infants with butyrate and propionate inhibit several inflammation-induced cytokines and chemokines (TNF-α, IL-6, IL-1β, CCL2, CCL8, CXCL5, CXCL8 and CXCL10) and prostaglandin (PTGS2) mRNA expression through suppressing activation of NF-_κ_B and mitogen-activated protein kinase (MAPK) signaling pathways [139].

In one experimental study, supplementation with *B. infantis* EVC001 strain resulted in decreased IL-1β and TNFα production in preterm infants [115]. An in vitro human model showed that probiotic *L. rhamnosus* GG attenuates fetal intestinal epithelial cell line H4 inflammatory responses by inhibiting TLR3 and TLR4 mRNA expression and *Salmonella Typhimurium* (*S. Typhimurium*)-induced TNF-α mRNA expression [140]. Another in vitro study demonstrated a potential inhibitory effect of *B. infantis* and *L. acidophilus* on TLR2/TLR4 mRNA expression and IL-1β/LPS-induced IL-6 and IL-8 mRNA expression in fetal immature enterocytes FHs74 [141]. Pretreatment of immature enterocyte H4 cells with *B. longum* supp *infantis* resulted in suppression of interleukin-1 receptor-associated kinase 2 (IRAK-2) and IL-1β-induced IL-6 and activator-protein 1 (AP-1) transcription factors c-Jun and c-Fos mRNA expression in a TLR-4-dependent manner [142]. It has also been shown that IL-1β-induced IL-8 mRNA expression is inhibited via downregulating the signal transducer and activator of the transcription 1 (STAT1) signaling pathway in immature enterocyte H4 cells pretreated with indole-3-lactic acid (ILA), a predominant breastmilk tryptophan metabolite, produced by *B. longum* supp *infantis* [143]. Treatment with the probiotic strains *B. infantis* and *L. acidophilus* modulates the inflammatory response of immature enterocyte H4 cells by inhibiting IL-1β-induced IL-6 and IL-8 mRNA expression and NF-κB p65 levels [144]. Two studies have shown that Polysaccharide (PSA) pretreatment produced by *B. fragilis* reduces inflammation in immature enterocyte H4 cells by reducing IL-1β-induced IL-8, CXCL5, CXCL10, matrix metalloproteinase-1 (MMPI), P-c-Jun and zona pellucida protein 4 (ZP4) mRNA expression in both TLR2 and TLR4 dependent-manner [145,146].

Taken together, these findings suggest that SCFAs and SCFA-producing bacteria may have a potential anti-inflammatory role in neonatal NEC by protecting fetal intestinal epithelial cells against pro-inflammatory cytokines and chemokines via inhibition of different cellular signaling pathways.

**Figure 1:**
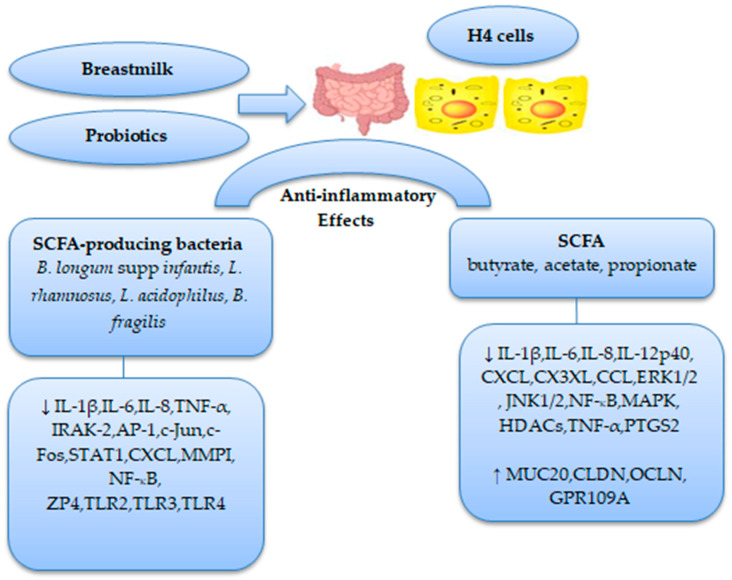
Role of SCFAs and its producing bacteria in neonatal NEC therapy. (**↓**) decrease; (**↑**) increase.

Figure 1 summarizes the therapeutic role of SCFAs and SCFA-producing bacteria in neonatal NEC.

## 7. Conclusions

SCFAs as epigenetic substrates perform a significant role in mediating microbe-host immune interactions, which could be a potential treatment for NEC-induced inflammation. During the colonization process in preterm infants, the gut is exposed to microbes that are more pathogenic but less commensal, which may contribute to NEC by increasing TLR4 signaling, leading to release of pro-inflammatory cytokines and chemokines. Breastmilk and/or formula with probiotics/prebiotics could modulate preterm infants’ gut microbiota colonization by decreasing the growth of pathogenic microbes, while increasing microbial species belonging to phyla Actinobacteria (*Bifidobacterium* spp.), Firmicutes (*Lactobacillus* spp., *Enterococcus* spp., *Streptococcus* spp.) and Bacteroidetes (*B. fragilis*), which produce different amounts of SCFAs.

SCFAs and SCFA-producing bacteria exert anti-inflammatory effects on cytokine and chemokine production in immature enterocyte H4 cells through inhibiting different signaling pathways. Breastmilk and feeding with probiotic/prebiotic formula increase SCFA production and the abundance of SCFA-producing bacteria in preterm infants. However, how these feeding types epigenetically determine NEC phenotype by exerting anti-inflammatory effects are still being unraveled. In conclusion, SCFAs and SCFA-producing bacteria could be potential targets in the treatment of NEC. Further studies are needed to examine whether breastmilk or feeding with probiotic/prebiotic formula could increase SCFA levels and influence the growth of SCFA-producing bacteria and the protective effects thereof on reducing NEC-related inflammatory markers through the epigenetic mechanisms.

## Figures and Tables

**Table 1:** Effects of feeding types on gut microbiota and its metabolite SCFAs bacteria in preterm infants.

Study Design	Sample Characteristics	Feeding Types	SCFAs/Microorganisms	References
Prospective cohort study	32 preterm infants delivered at <32 weeks of gestation over 1-year period	breastmilk with bovine milk-based fortifier OR Similac advanced special care formula	Breastmilk = acetate, propionate ↑ Formula = butyrate ↑	[109]
Prospective cohort study	60 preterm infants delivered at ≤36 weeks of gestation/birth weight (<1500 g) over 1-year period	Breastmilk OR formula	Breastmilk = propionate ↑ Formula = butyrate, acetate ↑	[110]
Prospective cohort study	234 preterm infants = 101 infants orally supplemented with probiotics over 100 day-period and 133 infants not supplemented	Probiotics *Bifidobacterium* and *Lactobacillus* (Bif/Lacto)	Bif/Lacto group = lactate, acetate, *B. bifidum*, *B. breve* ↑	[111]
Prospective cohort study	95 preterm infants delivered at <32 weeks of gestation who did not develop a disease over a study period of 10 years received probiotics and 28 infants not supplemented	Breastmilk, probiotic Infloran (*B. bifidum*, *L. acidophilus*), probiotic Labinic (*B. bifidum*, *B. longum* subsp. *infantis*, *L. acidophilus*)	Breastmilk = *Bifidobacterium* spp. ↑, *Staphylococci* ↓ Infloran probiotic= *B. breve*, *B. bifidum, E. faecium* ↑, *Veillonella parvula*, *Propionibacterium acnes* ↓ Labinic probiotic = *B. animalis* ↑ No probiotic = *Klebsiella* spp. ↑	[112]
Prospective cohort study	134 preterm infants delivered at <32 weeks of gestation/birth weight (<1500 g) over 1-year period	Breastmilk, OR probiotic formula (*L. acidophilus*, *L. bifidus*, *B. bifidum*)	Breastmilk = *Bifidobacterium* spp. ↑, *Veillonella* ↓ Probiotic formula = Lactobacillus ↓	[113]
Prospective cohort study	90 preterm infants delivered at <37 weeks of gestation/birth weight (<2500 g) fed with breastmilk or probiotic formula over 3 months and 48 infants received no probiotic	Breastmilk OR probiotic formula (*B. lactis*)	Probiotic = *Bifidobacterium*, *Lactobacillus* ↑ No probiotic = *Enterococcus*, *Streptococcus*, *Klebsiella* ↑	[114]
Prospective cohort study	31 preterm infants delivered at <32 weeks of gestation/birth weight (<1500 g) received probiotics over 5 month-period and 46 infants non-supplemented	Probiotic *B. infantis* EVC001	Probiotic group = Bifidobacteriaceae ↑, Staphylococcaceae, Enterobacteriaceae ↓	[115]
Prospective cohort study	42 preterm infants over 3 month-period	Breastmilk OR donor human milk	Breastmilk = *B. longum*, *B. breve*, *B. pseudolongum* spp. *globosum*, *B. longum* spp. *infantis*, *B. animalis* spp. *lactis*, *B. adolescentis*, *B. bifidum*, *B. dentium* ↑ Human donor milk = *B. bifidum*, *B. longum* spp. *longum*, *B. reuteri*, *B. vansinderenii*, *B. pseudolongum* spp. *pseudolongum*, *B. animalis* spp. *lactis*, *B. longum* spp. *suis* ↑	[116]
Prospective cohort study	117 preterm infants delivered at ≤32 weeks of gestation over 26-day period	Breastmilk, donor human milk OR formula	Breastmilk = *Bifidobacterium*, *Lactobacillus*, *Bacteroides*, *Bacteroidetes*, *Enterococcus* ↑	[117]
Prospective cohort study	29 preterm infants over 30-day period	Breastmilk	Clostridiales, Lactobacillales ↑	[118]
Prospective cohort study	12 preterm infants supplemented with a probiotic over 10 weeks-period and 10 infants not supplemented	Probiotic *B.breve* M-16V	Probiotic group = *Bifidobacterium*, *Enterococcus* ↑, *Lactococcus*, *Klebsiella*, *Rothia* ↓	[119]
Prospective cohort study	28 preterm infants supplemented with single or mixture probiotics over 6 month-period and 16 infants not supplemented	Single-strain probiotic *B.breve* M-16V Multiple-strain probiotics (*B. longum* subsp. *infantis* M-63, *B. breve* M-16V and *B. longum* subsp. *longum* BB536)	Single-strain probiotic = *Clostridium* ↓ Multiple-strain probiotics = bifidobacteria ↑, Enterobacteriaceae ↓	[120]
Prospective cohort study	31 preterm infants delivered at <32 weeks of gestation received probiotics over 4-month period, 35 preterm infants not received probiotics and 10 healthy full-term infants	Probiotic Infloran (*L. acidophilus* ATCC 4356, *B. longum* subspecies *infantis* ATCC 15697	Probiotic = *Bifidobacterium*, *Lactobacillus* ↑	[121]
Prospective cohort study	36 preterm infants delivered at birth weight (<1500 g) received two different probiotics over 5-month period, and 18 infants did not received probiotics	Probiotic *L. rhamnosus* Probiotics *B. infantis* and *L. acidophilus*	Probiotic groups = *Bifidobacterium*, *Lactobacillus* ↑	[122]
Prospective cohort study	8 preterm infants received a probiotic formula over 5-month period, and 14 infants not received a probiotic	Probiotic formula (*B. breve* HA-129, *B. bifidum* HA-132, *B. longum* subsp. *longum* HA-135, *B. longum* subsp. *infantis* HA-116, *L. rhamnosus* HA-111)	Probiotic group = *Bifidobacterium*, *Lactobacillus* ↑	[123]
Prospective cohort study	7 preterm infants delivered at <32 weeks of gestation received a probiotic formula over 1-month period and 3 preterm infants not received a probiotic	Probiotic Infloran (*B. bifidum* ATCC15696 and *L. acidophilus* NCIMB701748)	Probiotic group = *Bifidobacterium*, *Lactobacillus* ↑	[124]
Prospective cohort study	10 Preterm infants at delivered at <32 weeks of gestation received two different probiotics over 1-month period	Probiotics *B. longum* subsp. *infantis* OR *L. reuteri*	Probiotic *B. longum* subsp. *Infantis* = *Bifidobacterium* ↑ Probiotic *L. reuteri* = HMO ↑	[125]
Prospective cohort study	87 preterm infants at delivered at <30 weeks of gestation received a probiotic formula over 1-month period, and 165 infants did not receive a probiotic	Probiotic formula (*L. rhamnosus* GG and *B. animalis* ssp. *lactis* BB-12)	Probiotic group = Firmicutes, Actinobacteria ↑, *Weissella*, *Veillonella*, *Klebsiella* ↓	[126]
Prospective cohort study	70 preterm infants at delivered at ≤28 weeks of gestation received a probiotic formula over 1-month period, and 50 infants did not receive a probiotic	Probiotic Infloran (*L. acidophilus* and *B. bifidum*)	Probiotic group = *Lactobacillus* spp., *E. faecium* ↑, Yersiniaceae, *Staphylococcus*, *Klebsiella* spp. ↓	[127]
RCT	90 preterm infants delivered at <35 weeks of gestation supplemented with either probiotic species (CUL) or prebiotic/probiotic combinations (PBP) 29 preterm infants received Pregestamil formula (placebo)	CUL probiotics = Two *lactobacillus* spp. PBP probiotics = Several *lactobacillus* and *Bifidobacterium* spp. plus fructo-oligosaccharides	PBP group = acetate, bifidobacteria ↑ Placebo = bifidobacteria ↓	[128]
RCT	173 preterm infants delivered at <28 weeks of gestation supplemented with either single (SS) or triple-strain (TS) probiotics 29 preterm infants (no probiotics, placebo)	SS probiotic = *B. breve* M-16V TS probiotics = *B. breve* M-16V, *B. longum* subsp. *infantis* M-63 and *B. longum* subsp. *longum* BB536	SS group = propionate, *B.breve*, *B.bifidum* ↑, *Clostridium* ↓ TS group = butyrate, propionate, *B. longum*, *B. reuteri*, *B. longum* subsp. *infantis*, *B. longum* subsp. *longum* ↑, *Clostridium* ↓ Placebo = *Clostridium butyricum*, *Streptococcus salivarius*, *S. thermophilus* ↑	[129]
RCT	17 preterm infants delivered at <31 weeks of gestation/birth weight (<1500 g) supplemented with probiotic (Bifid) 18 preterm infants received vehicle supplement only (placebo)	Probiotic *B. breve* BBG-01	Bifid group = *Bifidobacterium* ↑	[130]
RCT	15 preterm infants delivered at <32 weeks of gestation supplemented with prebiotic formula 15 preterm infants supplemented with maltodextrin (placebo)	Prebiotic formula (mixture of fructo-oligosaccharides and galacto-oligosaccharides)	Prebiotic group = bifidobacteria ↑	[131]
RCT	38 preterm infants delivered at <32 weeks of gestation/birth weight (<1500 g) supplemented with probiotics mixture 28 preterm infants supplemented with maltodextrin (placebo)	Probiotics mixture (*S. thermophilus* TH-4, *B. longum* subsp. *infantis* BB-02 and *B. animalis* subsp. *lactis* BB-12)	Probiotics group = *Bifidobacterium* ↑	[132]
RCT	37 preterm infants delivered at <37 weeks of gestation received a probiotic 32 preterm infants received Nestle ’ FM 2000B formula (placebo)	Probiotic *B. lactis* Bb12	Probiotic group = bifidobacteria ↑, Clostridia, *Enterobacteria* spp. ↓	[133]
RCT	54 preterm infants received a probiotic, and 54 infants no received any probiotic (placebo)	Probiotic *L. reuteri* DSM 17938	Probiotic group = *Lactobacillus* ↑, *Clostridium*, Enterobacteriaceae, Staphylococcaceae ↓	[134]
RCT	229 preterm infants delivered at <32 weeks of gestation received a probiotic formula, and 230 infants did not receive a probiotic (placebo)	Probiotic formula (*S. thermophilus* TH-4, *B. animalis* subsp. *lactis* BB-12 and *B. longum* subsp. *infantis* BB-02)	Probiotic group = *B. longum* subsp. *infantis*, *B. animalis* subsp. *lactis*, *S. thermophilus* ↑	[135]

(↓) decrease, (↑) increase.

## Data Availability

Not applicable.
